# Characterization of the Airflow within an Average Geometry of the Healthy Human Nasal Cavity

**DOI:** 10.1038/s41598-020-60755-3

**Published:** 2020-02-28

**Authors:** Jan Brüning, Thomas Hildebrandt, Werner Heppt, Nora Schmidt, Hans Lamecker, Angelika Szengel, Natalja Amiridze, Heiko Ramm, Matthias Bindernagel, Stefan Zachow, Leonid Goubergrits

**Affiliations:** 1 Institute for Imaging Science and Computational Modelling in Cardiovascular Medicine, Charité - Universitätsmedizin Berlin, Berlin, Germany; 2 Department of Otorhinolaryngology, Head and Neck Surgery, Städtisches Klinikum Karlsruhe, Karlsruhe, Germany; 3 Department of Otorhinolaryngology, Head and Neck Surgery, Parkklinik Weißensee, Berlin, Germany; 4 1000shapes GmbH, Berlin, Germany; 5 Department of Visual Data Analysis - Zuse Institute Berlin (ZIB), Berlin, Germany; 6 Einstein Center Digital Future, Berlin, Germany

**Keywords:** Medical research, Respiratory signs and symptoms, Biomedical engineering, Computational science

## Abstract

This study’s objective was the generation of a standardized geometry of the healthy nasal cavity. An average geometry of the healthy nasal cavity was generated using a statistical shape model based on 25 symptom-free subjects. Airflow within the average geometry and these geometries was calculated using fluid simulations. Integral measures of the nasal resistance, wall shear stresses (WSS) and velocities were calculated as well as cross-sectional areas (CSA). Furthermore, individual WSS and static pressure distributions were mapped onto the average geometry. The average geometry featured an overall more regular shape that resulted in less resistance, reduced WSS and velocities compared to the median of the 25 geometries. Spatial distributions of WSS and pressure of the average geometry agreed well compared to the average distributions of all individual geometries. The minimal CSA of the average geometry was larger than the median of all individual geometries (83.4 vs. 74.7 mm²). The airflow observed within the average geometry of the healthy nasal cavity did not equal the average airflow of the individual geometries. While differences observed for integral measures were notable, the calculated values for the average geometry lay within the distributions of the individual parameters. Spatially resolved parameters differed less prominently.

## Introduction

The nose is the main passageway for respired air to flow from ambient to the lungs and vice versa. Air flowing through the nose is humidified, tempered and cleansed from particles, which could harm the intricate structures of the lungs. While the anatomy of the nose can easily be investigated from the outside using either a speculum or an endoscope, the nature of healthy nasal airflow is not yet fully understood. Nonetheless, there were extensive efforts as well as remarkable progress to better understand the complex airflow within the nose.

While investigation of the airflow within the nasal cavity began with *in-vitro* experiments using either cadaver castings or upscaled models^1–3^, numerical simulation of nasal airflow became the quasi standard within the last decade and is now widely used^4–6^. Here, the patient-specific geometry of the nasal cavity is reconstructed using computed tomography (CT) scans.

Recently, first studies were able to reveal correlations between numerically calculated flow parameters and the perceived nasal patency of a patient. Zhao *et al*. were able to show, that a significant correlation between cooling of the mucosal layer, a process that is associated with trigeminal function, is correlated with patency ratings of the nose^7,8^. In more recent studies, they were able to reveal differences in wall shear stress and heat flux between symptomatic and asymptomatic patients with septal perforations^9^ and they were also able to show, that numerical simulations might help to understand complex relationships between surgical procedures and the development of empty nose syndrome^10^. Sanmiguel-Rojas *et al*. proposed another approach, where two non-dimensional parameters, one related to the nasal shape, the other to the nasal resistance, are used to differentiate between healthy and disturbed nasal breathing^11^.

Even though impressive progress was made towards understanding the airflow in the nose and even first methods for discrimination of healthy and disturbed nasal airflow were proposed, there is still no clear definition of what constitutes healthy airflow.

One issue that impedes progress in this field is the large heterogeneity of the human nasal cavity and the resulting aerodynamic variance even in healthy subjects^12,13^. The human nasal cavity is not only an extremely complex geometry, but its individual features differ grossly even if the main anatomical structure remains similar. Even within a single patient, the morphology of the nasal cavity changes periodically with time and external conditions^14,15^. However, a lot of *in-vitro* and in-silico investigations are performed using individual geometries. The heterogeneity makes it difficult to compare findings made using different geometries, be it between different research groups, but also within a sample.

One approach to overcome the problem of limited comparability when patient-specific geometries are used is the generation of synthetic geometries. Ideally, such a geometry is an average of the overall shape variance observed within a patient population. Liu *et al*. already generated such an average geometry of the healthy nasal cavity in 2009^16^ by registration and subsequent superposition of CT image data of 30 patients. The resulting geometry was titled “Carlton-Civic Standardized Nasal Model” and was then proposed as geometric standard for *in-vitro* and numerical investigations as well as baseline for comparison against patient-specific geometries. Comparison of this average geometry against the individual geometries used for its generation showed good agreement with respect to cross-sectional areas. However, the three-dimensional shape varied visually from the shape commonly observed. While the key features of the three meatus were preserved, the isthmus nasi, the narrowest cross section that often features a distinct notch at the anterior part of the nose, was smoothed out during the averaging procedure.

Using two other techniques, identification of medial axes of the nasal cavities cross-sections as well as describing those cross-sections using a Fourier series, Gambaruto *et al*. generated average geometries of three subjects^17,18^. Both approaches allow compact representation of the complex geometry of the nasal cavity and subsequent averaging of multiple geometries. The geometries investigated by Gambaruto *et al*. featured all commonly observed anatomical characteristics. Furthermore, comparison of the simulated aerodynamics revealed good agreement with the airflow observed in the three individual geometries used for averaging. However, the nasal resistance as well as wall shear stresses were reduced in the average model compared to the three individual geometries.

Such approaches describing a complex shape using a compact representation are often described as statistical shape modeling (SSM). While this term describes a large set of different techniques, the underlying approach of those is similar: the geometry is represented in a reduced manner (reduced basis) that allows to quantify the observed variance in different shapes. An SSM of the nasal cavity that was generated using image data of 46 patients was presented by Keustermans *et al*.^19^. They were able to demonstrate, that generation of a statistical shape model of the human nasal cavity is feasible. The resulting average geometry featured all relevant landmarks and even a distinct isthmus nasi was observed.

Even if a representative average geometry of the nasal cavity can be generated which is commonly used, one problem remains. Comparison of flow information measured or calculated within individual geometries against information obtained for the averaged geometry might still be difficult. The reason for this is the large heterogeneity of the nasal cavity’s anatomy. Inthavong *et al*. proposed a method to overcome this problem. Here, the complex and tortuous three-dimensional geometry of the nasal cavity’s wall is projected onto a rectangular plane in two dimensions^20^. Thus, wall-bound information as the static pressure and the wall shear stress can be projected onto a simpler geometry. Using such a dimensional reduction approach, parameter distributions of different patients can be compared much easier^21^. While this approach does not necessarily map the same semantic region of two different patients onto the same coordinates of the plane, an updated approach was proposed that allows an identification of relevant regions, such as the olfactory cleft^22^.

In this study, we also generated a statistical shape model of the health human nasal cavity from 25 patient-specific samples segmented from medical images. An averaged geometry was derived from our statistical shape model, which is made openly available and will be constantly extended and updated in the future (see data availability section). However, the relationship between airflow and geometry is described by non-linear partial differential equations: the Navier-Stokes-Equations. Thus, the question was whether the airflow within an averaged geometry of the healthy human nasal cavity equals the average of the airflow observed in individual geometries. While a standardized geometry might be helpful for comparison of different studies against each other, such a geometry’s use is limited if the airflow observed within it is a poor representation of the average healthy nasal airflow.

The patient-specific airflow was calculated for all 25 patient-specific geometries as well as for the average geometry generated using the SSM. As the approach used for generation of the SSM resulted in a common surface discretization for all geometries included within the SSM, a point-wise comparison of the flow fields observed in patient-specific geometries and the mean geometry became possible. Using this approach, we wanted to investigate whether the airflow calculated for the average geometry was similar to the average airflow calculated for all individual cases.

## Materials and Methods

### Patient and image data

For this retrospective investigation anonymized computed tomography (CT) data of 25 symptom-free subjects that were treated in the Ear, Nose and Throat practice run by one of the authors was used. Due to the retrospective nature of this study, the 3D CT image data was acquired using different devices and had a voxel resolution of 0.37 ×0.37 ×0.4 mm³ or better. Indications for the CT scans were facial pain, hyposmia or anosmia, unilateral swelling of an eyelid, suspicion of recurrent sinusitis, possible cerebrospinal fluid leak, recurrent epistaxis and epiphora. The patients’ age ranged from 17 to 57 years and was 37 years on average. Six of the patients were male. Patients were only included in this study if they reliably stated unimpaired nasal breathing. Furthermore, no severe alterations of the nasal anatomy or chronic diseases, as for example septal perforations, were observed upon investigation by an ENT specialist. Neither rhinoscopy and endoscopy nor the CT scans revealed pathological findings that could have compromised nasal breathing. The actual reasons for the consultations were not related to breathing complaints.

The study was carried out according to the principles of the Declaration of Helsinki and approved by the local ethics committee (Ethics committee - Charité - Universitätsmedizin Berlin). Written informed consent was obtained from the participants.

### Image segmentation

Reconstruction of the patient-specific geometry of the nasal cavity from the 3D CT image data was performed using semi-automated segmentation tools provided with ZIBAmira (v. 2015.28, Zuse Institute Berlin, Germany). Image voxels with a Hounsfield Unit (HU) above −400 were masked as tissue. Thus, only voxels below this threshold were considered as candidates for the segmentation of the nasal cavity^23,24^.

Then, the image data was segmented slice by slice, beginning from the frontal coronal slice (from anterior to posterior). Both sides of the nasal cavity were segmented using a flood fill tool, that connected all contiguous regions. All regions belonging to the nasal meatus or the main passageway parallel to the nasal septum were selected. Neither the sinuses nor the ethmoidal air cells were included within the segmentation. Afterwards, the segmentation was corrected by slicing through the data stack from top to bottom as well as from left to right.

As all geometries had to feature the same topology, small holes in the segmentation that resulted from narrow passageways or weakly contrasted regions had to be closed manually. Furthermore, all artificial connections between the three meatus or both sides of the nasal cavity, usually resulting from those structures being separated only by very thin tissue, had to be removed. This step was necessary in nine geometries. The affected regions were all located in the posterior, superior part of the nasal septum and resulted from slight deviations of the septal wall in this area. In the most extreme case, this correction resulted in a change of the number of voxels included in the segmentation by 0.2 percent. In every case, the patency of the nose in the affected region was re-affirmed by the ENT specialist.

**Figure 1:**
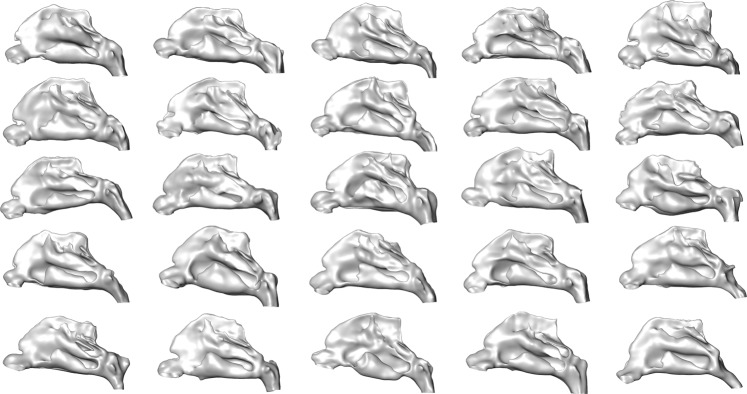
Visualization of all 25 individual nasal cavity geometries.

Reconstructed geometries were then smoothed using a volume-preserving smoothing algorithm^25^. To ensure that smoothing of the geometries did not result in severe alterations of the segmented geometries, original and smoothed surfaces were compared visually, as well as using the mean distance and the Hausdorff distance between the unaltered and the smoothed geometry. The latter is defined as the maximum value of all minimal distances between the two geometries. Therefore, for each vertex of the unaltered geometry the minimal distance to the smoothed geometry was calculated. The maximum of those distances is the Hausdorff distance. Mean distances were below 0.12 mm for all geometries, which is below the voxel resolution used for image segmentation. The highest Hausdorff distance calculated was 1.59 mm. While this equals four times the voxel resolution used for image segmentation, it is the maximal deviation observed between all geometries. Large distances between two geometries were only observed in regions with high curvature, such as the superior terminations of the meatus. All segmented geometries are shown in Fig. 1.

### Statistical shape analysis

A statistical shape model (SSM) of the nasal cavity was created to achieve a common triangulation of all patient-specific geometries as well as to allow generation of an average geometry of all 25 subjects. To virtually enhance the data for generation of the SSM, also the axially mirrored geometries were used, which is a common approach^19^.

**Figure 2:**
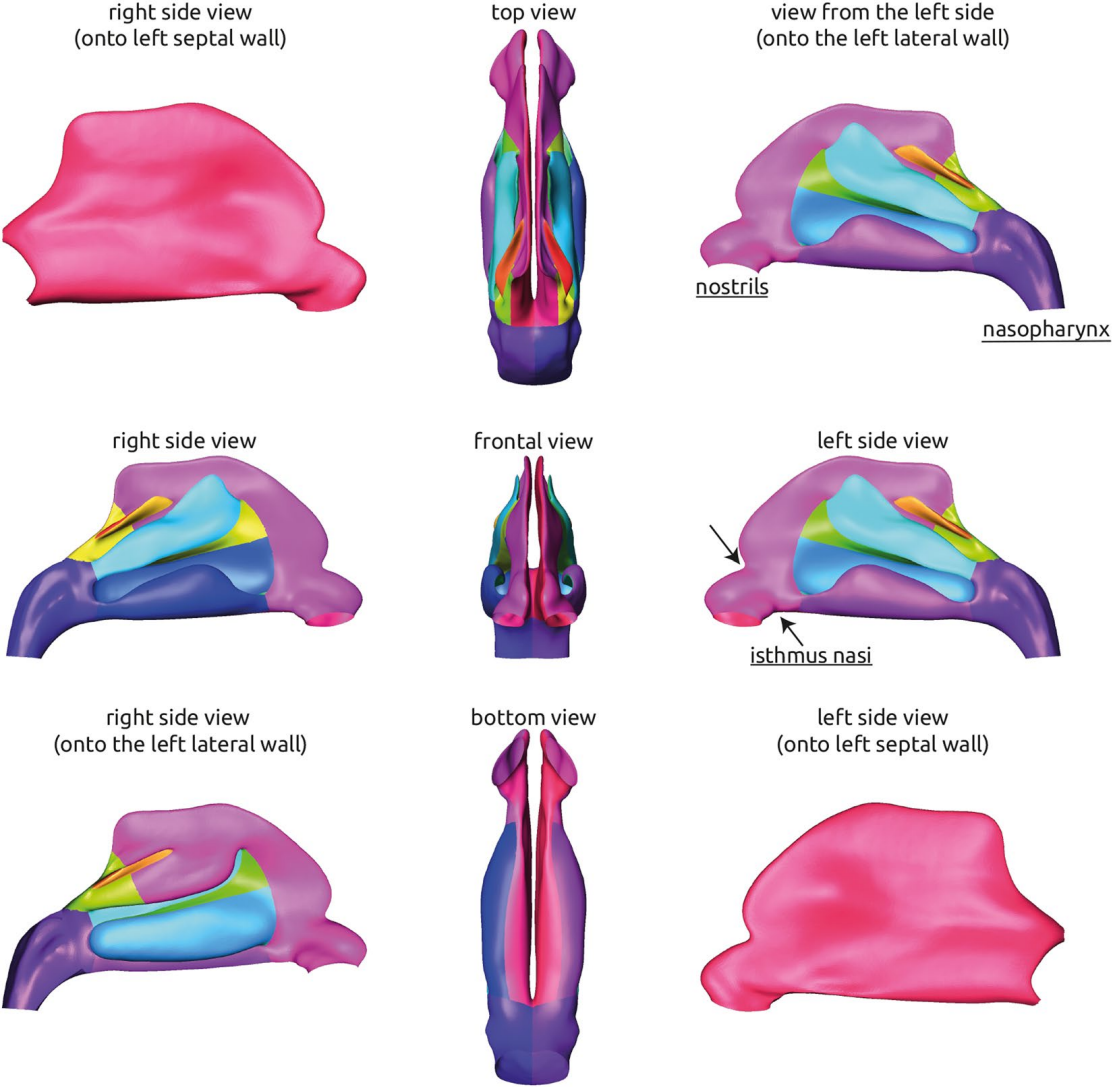
Visualization of the average geometry of the healthy nasal cavity generated using the statistical shape model. The geometry is shown from different angles. The corresponding 24 patches that were identified for each geometry and used for subsequent generation of the statistical shape model are visualized using separate colors.

Using a method presented by Lamecker *et al*.^26,27^, the smoothed surfaces were manually subdivided into 24 so called patches (12 per nasal cavity), anatomically corresponding subregions like the meatus, the septal wall, or the pharynx. An illustration of all patches is given in Fig. 2. The patches identified for all geometries were approved by an ENT specialist.

**Figure 3:**
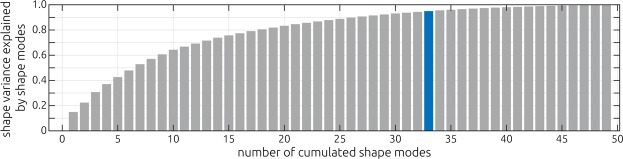
Visualization of the shape variance explained by the different shape modes of the statistical shape model. The amount of the overall shape variance observed in the 50 individual geometries is plotted against the number of cumulated shape modes. The highlighted bar (n = 33) indicates the number of shape modes that are required to explain 95 percent of the shape variance.

Point-to-point correspondences were then established on the patched surfaces using the method described earlier^28^. Performing principal component analysis (PCA) on the corresponding point clouds yielded an SSM that contains the average shape of the nasal cavities as well as the typical geometric variation occurring in the input population. The relative ratio of variance in the observed shapes, that is explained by the different shape modes identified using the PCA is shown in Fig. 3. The average geometry of the healthy nasal cavity is then calculated as the mean of all shape modes and their respective weights.

### Numerical simulation

The common triangulation of all 25 geometries, which was generated during the statistical shape analysis, was not sufficient for CFD simulations, as the average mesh size of 0.5 mm was too coarse, and the common triangulation of all geometries led to triangular cells with large aspect ratios in some cases. Therefore, a finer surface mesh had to be generated. Numerical meshes were generated using the polyhedral meshing tool provided by STAR-CCM+ (v. 13.02, Siemens PLM, USA). A base size of 0.3 mm was specified for the entire geometry. The remeshing procedure did not alter the surface geometry. To correctly capture the near wall flow, five boundary layers with increasing thickness were introduced. The thickness of each subsequent layer was 20 percent larger than that of the previous layer. The boundary layer’s overall thickness was 0.2 mm. This mesh resolution was shown to yield mesh independent results^29,30^.

Numerical simulation of the patient-specific flow fields within the 25 patient-specific nasal cavity geometries as well as the mean geometry was performed using STAR-CCM+. Restful inspiration and expiration were simulated using a quasi-steady assumption. At the truncated nostrils a constant, relative static pressure of 0 Pa was assumed. At the truncated pharynx a constant velocity boundary condition was used. This velocity was set to a value that resulted in a volume flow rate $\dot{V}$ of 200 ml/s, equaling a respiratory minute volume of 6 liters. Since Mach numbers of all simulations were small (<0.01), respired air was modelled as incompressible fluid with a constant density of 1.18 kg/m³ and a constant dynamic viscosity of 18.6 μPa·s. As only slow, restful breathing was simulated, laminar flow was assumed^24^. The nasal cavities’ walls were assumed to be rigid and a no-slip boundary condition was applied.

For all 25 patient-specific geometries as well as the mean geometry generated using the statistical shape model, wall shear stresses and static pressures at the nasal cavities’ wall were exported.

### Analysis

As described previously, the common triangulation of the statistical shape model was not sufficient for the numerical simulations. Therefore, a new surface mesh with a finer and more consistent resolution had to be generated. However, this remeshing only affected the triangulation, the surface boundary was not altered in this process. As the resolution of the volumetric meshes being used for simulation was finer than that of the statistical shape model and the shape was not altered, wall-bound information calculated using numerical simulations could be mapped onto the original triangulation of the SSM. To facilitate this, static pressure and wall shear stress distributions at the nasal cavities’ wall were imported in MATLAB (v. 2018a, The Mathworks Inc., USA). Here, a nearest interpolation scheme provided by the function *scatteredInterpolant* was used to map calculated data from the numerical grid onto the coarser triangulation of the reference model triangulation.

Using this approach, wall shear stress and static pressure information during inspiration and expiration were mapped onto the original triangulation of the SSM. As all geometries included in the SSM shared the same triangulation, vertex-wise medians and standard deviations of the static pressure and wall shear stresses were calculated. These were then compared against values calculated for the mean geometry generated using the SSM.

Additionally, volume- as well as surface-averaged aerodynamic parameters were calculated for all patient-specific geometries as well as the mean geometry. These include the volume-averaged velocity, the maximal velocity observed within the nasal cavity, the surface-averaged wall shear stress, the airflow partitioning, which is defined as the ratio between the unilateral airflow in the left nasal cavity and the bilateral airflow, the lateral nasal resistances $({R}_{left/right}=\Delta p/{\dot{V}}_{left/right})$ and the total nasal resistance $({R}_{total}=({R}_{left}\cdot {R}_{right})/({R}_{left}+{R}_{right}))$. Velocities and resistances were calculated, since the current state of the presented SSM does allow point-wise comparison of only wall-bound information but does not yet allow comparison of volumetric information as for example the intranasal flow field. As those additional parameters are not spatially resolved but are calculated by averaging, they will be referred to as integral measured to allow better differentiation with the spatially resolved parameters.

Additionally, the cross-sectional area perpendicular to inspiratory streamlines were calculated using a method described by Garcia *et al*.^31^. For each subject, five streamlines were generated for each side of the nose using STAR-CCM+. Using ZIBAmira, cross-sections were calculated perpendicular to each streamline. This evaluation was performed from the beginning of the nostril towards the choane. Then, the median of the calculated cross-sectional areas was calculated for each subject and side.

## Results

### Mean geometry and the patches

The average geometry that was generated using the statistical shape model is shown in Fig. 2. Here, all 24 patches that were identified for all 25 individual geometries during generation of the SSM are highlighted using different colors. The position of the nostrils and the nasopharynx is indicated as well as the position of the isthmus nasi, which features the narrowest cross section of the nasal cavity. The average geometry features all common anatomical landmarks, that are usually observed in a healthy nasal cavity. All three meatus, that are formed by the nasal conches are present. With the superior meatus being the smallest and the inferior meatus being the largest. Due to mirroring of the training data that was used for generation of the statistical shape model, the average geometry is perfectly symmetric (see Fig. 2). The distinct shape of the isthmus nasi, which features a clear notch in the anterior part of the nasal cavity was preserved after averaging. The openings of the nostrils are directed slightly lateral.

In Fig. 3, the shape variance that is explained by the shape modes identified using the principle component analysis is visualized. The figure shows, how many cumulated shape modes are necessary to identify a specific amount of the shape variance observed in the geometries that were used to generate the statistical shape model. Here, the modes are sorted in a decreasing way, meaning that the first shape mode explains most of the variance observed in the sample. The first 33 modes were necessary to explain 95 percent of the shape variance observed in the sample.

### Comparison of the cross-sectional area

**Figure 4:**
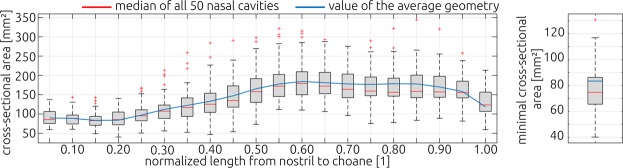
Comparison of the cross-section area along the length of the nasal cavity (left). Beginning from the nostril towards the choane, cross-section areas were calculated for each individual cavity. The box plots illustrate the distribution observed for all 50 cavities (25 subjects, left and right side). The red lines indicate the median value of each distribution. The blue line illustrates the cross-section areas calculated for the average geometry generated using the statistical shape model. The distribution of the minimal cross-section areas observed for all 50 cavities is shown in the right panel. Here, the blue bar represents the value calculated for the average geometry.

Figure 4 shows the cross-sectional areas calculated for all subjects and the average geometry as well as the minimal cross section. The cross-sectional areas are plotted against the normalized distance from nostril to choane. For each 5 percent increment a box plot is shown, indicating the median value (red line) of the 50 individual nasal cavities (25 subjects, 2 sides) as well as the respective distribution. In the anterior half of the nasal cavity, the cross-sectional area calculated for the average geometry agrees well with the median values calculated for the individual geometries. In the posterior half, the values calculated for the average geometry deviate slightly from the median values of the individual geometries.

The minimal cross-sectional area of the average geometry is larger than the median of the individual geometries. It lies close to the 75-percentile of the individual values.

### Comparison of integral measures

**Figure 5:**
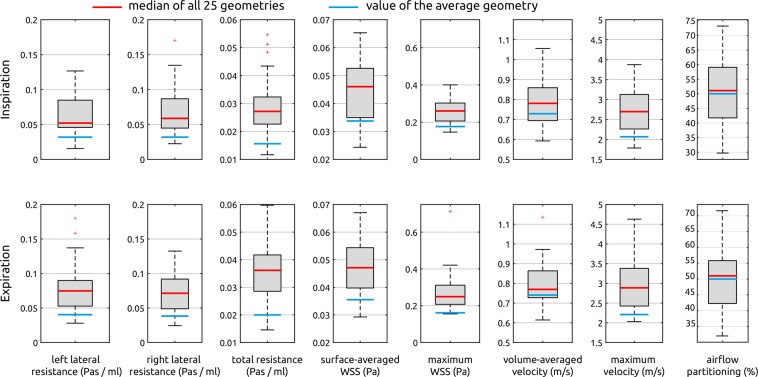
Comparison of integral measures calculated for the average geometry generated using the SSM as well as all 25 individual geometries. These parameters include the left and right nasal resistance as well as the total nasal resistance, the volume-averaged velocity, the surface-averaged wall shear stress, the 99.9 percentiles of the velocity and wall shear stress as well as the airflow partitioning, which is defined as the ratio between the unilateral airflow in the left nasal cavity and the bilateral airflow. Parameters calculated during inspiration and expiration are shown in the upper and lower row respectively. The median of all 25 individual simulations is highlighted as red bar, while the value calculated for the average geometry is highlighted using a blue bar.

The distribution of integral measures that were calculated for the 25 individual geometries as well as the values calculated for the average geometry generated using the SSM are shown in Fig. 5 using boxplots. Here, both lateral as well as the total nasal resistance, the volume-averaged velocity and surface-averaged wall shear stress, the 99.9% percentiles of the velocity and the wall shear stress as well as the airflow partitioning are shown for inspiration and expiration respectively. In general, all values calculated for the average geometry are below the median calculated from the individual geometries. Only in case of the volume-averaged velocity and the airflow partitioning, the value calculated for the average geometry lies between the 25% and 75% percentile of the distribution of all individual geometries.

On average, the total resistance of the patient-specific geometries was 33% larger during expiration than during inspiration. Almost the same ratio was observed for the average geometry, where the increase in resistance was 31%. Mean, median and standard deviation values of those distributions are specified in Table 1, all individual values are provided as supplemental material.

**Table 1:** Mean, median and standard deviation (SD) values calculated for all integral parameters as well as the average geometry generated using the statistical shape model (SSM). The left and right lateral as well as the total resistance (R), the surface-averaged and maximum wall shear stress (WSS), the volume-averaged and maximum velocity and the airflow partitioning (AP) are specified for inspiration and expiration respectively.

	Inspiration								Expiration							
	R [Pa·s/ml]			u [m/s]		WSS [Pa]		AP [%]	R [Pa·s/ml]			u [m/s]		WSS [Pa]		AP [%]
	left	right	total	avg.	max.	avg.	max.		left	right	total	avg.	max.	avg.	max.	
mean	0.063	0.069	0.030	0.78	2.77	0.044	0.265	50.26	0.079	0.071	0.036	0.80	2.99	0.047	0.277	50.29
median	0.052	0.059	0.027	0.78	2.70	0.046	0.260	51.13	0.075	0.071	0.036	0.77	2.89	0.047	0.249	50.97
SD	0.028	0.035	0.010	0.11	0.59	0.011	0.069	11.38	0.038	0.027	0.013	0.12	0.67	0.011	0.114	10.03
SSM	0.031	0.031	0.016	0.73	2.06	0.034	0.161	50.0	0.041	0.041	0.020	0.74	2.20	0.035	0.176	50.0

### Comparison of individual and averaged wall-bound information

**Figure 6:**
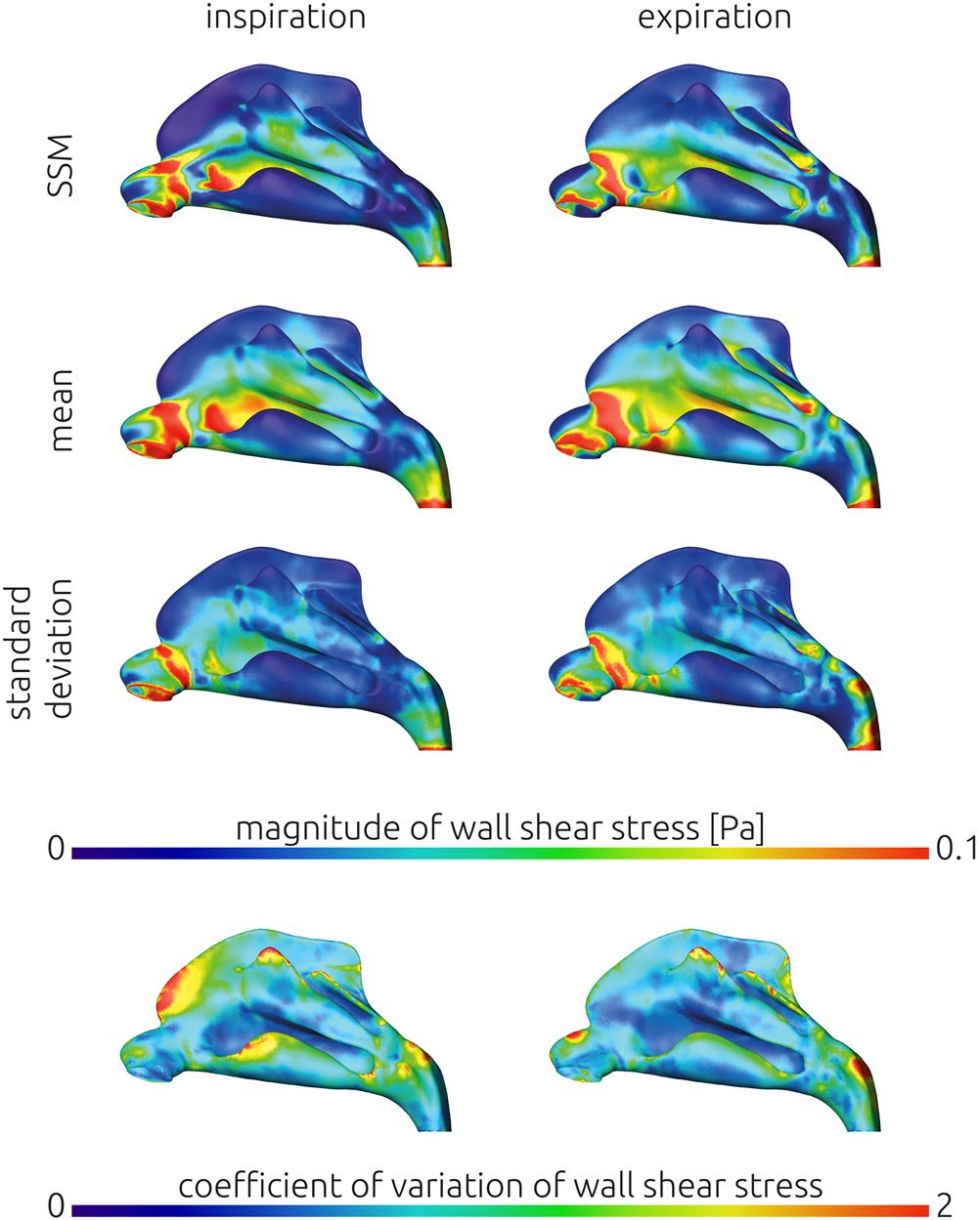
Comparison of the wall shear stress distribution calculated for the average geometry generated using the statistical shape model (SSM, upper row) against the mean distribution of wall shear stresses that was calculated from the 25 individual simulation results mapped onto the average geometry (second row). The standard deviation of those 25 simulation results is indicated in the third row to identify regions of relatively large variations. In the left column results for inspiration are shown, while the results calculated for expiratory airflow are shown in the right column. In the last row, the coefficient of variation (ratio of standard deviation and mean) is shown for the wall shear stress during inspiration and expiration.

Using the wall shear stress information of each subject that was mapped onto the average geometry, mean distributions were calculated for inspiration and expiration respectively. These are shown in Fig. 6 together with the corresponding WSS distribution calculated using the average geometry. Furthermore, the standard deviation of the wall shear stress distribution of all 25 subjects is shown.

Interestingly, the mean wall shear stress distribution agrees well compared against the distribution calculated using the average geometry. The images in Fig. 6 showing the wall shear stresses at the lateral wall of the nasal cavity during inspiration indicate two regions of elevated wall shear stress magnitudes (WSS > 0.1 Pa), one being the isthmus nasi and one lying directly behind the isthmus nasi between the lower and middle meatus. While both regions are present in the mean distribution as well as the distribution calculated using the average geometry, their shape differ. The mean distribution features overall larger areas that are exposed to wall shear stress magnitudes above 0.1 Pascal, while the average geometry’s distribution reveals a narrow band of elevated wall shear stresses. Furthermore, a jet like formation of the wall shear stresses can be observed during inspiration in the average geometry. This region relates to the large stationary vortex often observed in the upper, anterior part of the nose^32,33^. Here, the inspiratory jet detaches due to the relatively sudden increase of the cross-sectional area of the nasal cavity in distal direction, creating a shear layer.

During expiration, there is also a larger area that is exposed to elevated wall shear stresses in the mean distribution of all individual geometries compared to the average geometry’s distribution. In both distributions higher wall shear stresses are present in the region of the lower meatus. This correlates well with the common observation, that the nasal cavity is more evenly perfused during expiration than during inspiration, leading to higher velocities in the lower meatus.

**Figure 7:**
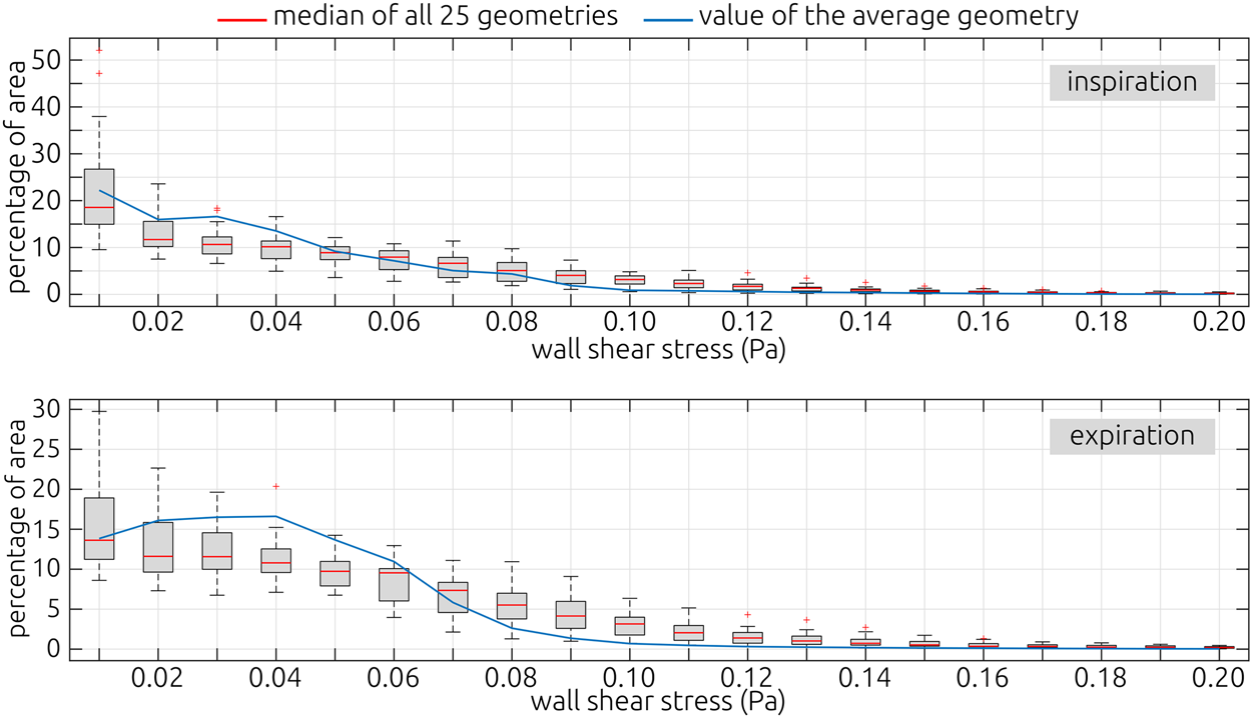
Histograms illustrating the percentage of surface area that is affected by different levels of wall shear stresses. The box plots indicate the distribution of affected surface areas calculated for the 25 patient-specific geometries. The red lines indicate each level’s median value. The blue line is illustrating the respective values calculated for the average geometry that was generated using the SSM. The upper panel illustrates the wall shear stress histograms during inspiration, while the lower panel illustrates the expiratory phase. The numbers below each box plot indicate the respective histogram bin’s upper limit (i.e. the first bin includes all wall shear stresses above 0 and below 0.01 Pa).

To better resolve these wall shear stress distributions, surface histograms of the wall shear stress magnitudes were calculated (Fig. 7). Here, the percentage of the surface area of the individual geometries affected by distinct levels of wall shear stresses are shown using box plots. The individual geometries’ median value is shown as well as the value calculated for the average geometry. The histograms further indicate, that for the individual geometries a larger portion of the surface is affected by higher wall shear stresses than for the average geometry.

During inspiration as well as expiration, the largest standard deviations were calculated within the isthmus nasi. This is to be expected, as the air reaches its maximum velocity in this narrow cross section. Thus, the wall shear stresses will be highest in this region. To better visualize the deviations, the coefficient of variation (ratio of standard deviation and mean) were also shown in Fig. 6. The coefficient of variation is more evenly distributed. While the values observed within the isthmus nasi are still larger than that in the posterior part of the nose, the differences are less prominent. Large relative deviations are only observed in areas with wall shear stresses close to zero (low signal to noise ratio).

**Figure 8:**
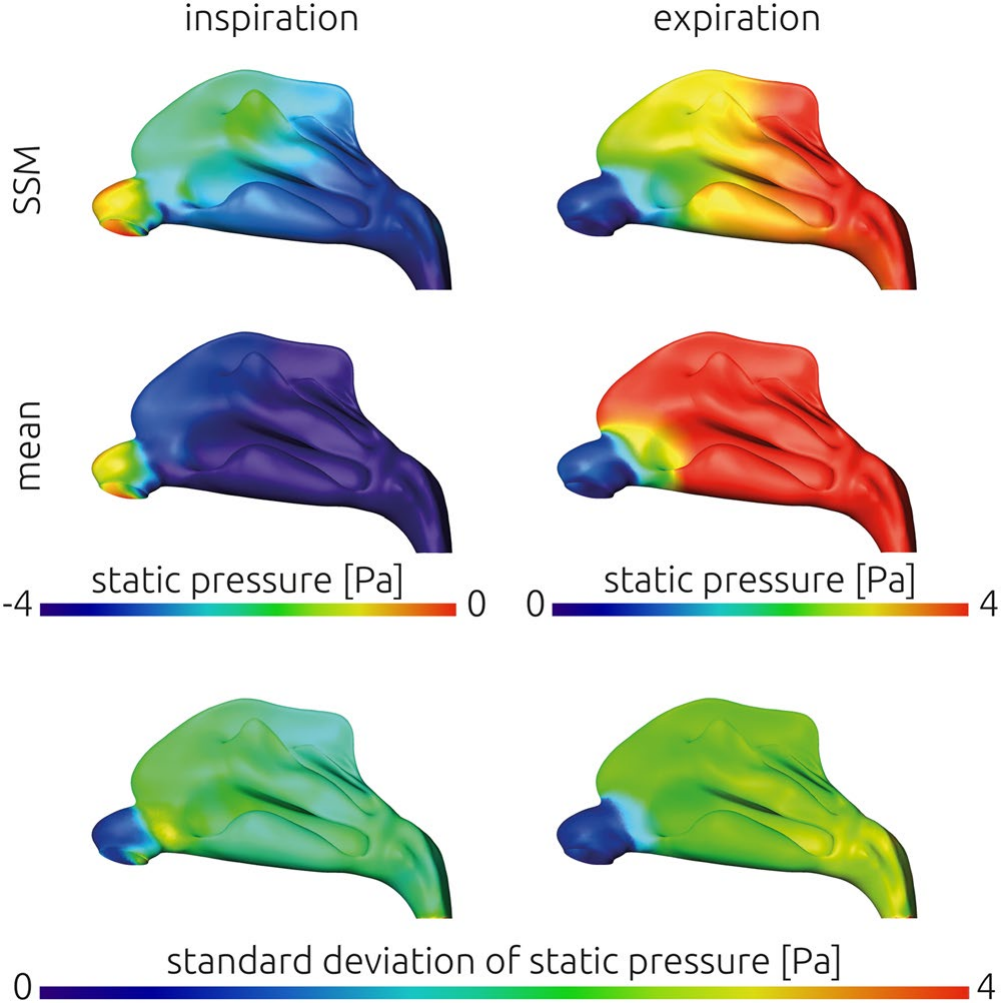
Comparison of static pressure distribution calculated for the average geometry generated using the statistical shape model (SSM, upper row) against the mean distribution of static pressure that was calculated from the 25 individual simulation results mapped onto the average geometry (middle row). The standard deviation of those 25 simulation results is indicated in the lower row to identify regions of relatively large variations. In the left column results for inspiration are shown, while the results calculated for expiratory airflow are shown in the right column.

Equivalent to the wall shear stresses, distributions of the static pressure are shown in Fig. 8. Here, the mean generated from the 25 individual distributions, the distribution calculated using the average geometry as well as the standard deviation of all 25 individual distributions are shown.

The overall pressure drop in the mean static pressure distribution calculated from the 25 individual results is larger, than that of the mean geometry during inspiration as well as expiration. This agrees with the observations regarding the integral measures. The maximal pressure gradient is observed at the isthmus nasi in the average geometry’s distributions as well as the mean distributions.

## Discussion

In this study we investigated an average geometry of the healthy nasal cavity and compared the numerically calculated airflow within this geometry against the averaged airflow observed within 25 patient-specific geometries. The main question of this investigation was: Does the airflow observed in an averaged geometry equal the average airflow observed within the patient-specific geometries? Additionally, we wanted to answer the question, whether the average geometry could be disseminated and used as a generic geometry for investigations regarding healthy nasal airflow.

Comparison of integral measures of the nasal resistance, wall shear stresses and air flow velocities revealed, that all those parameters were below the median values calculated using the 25 individual geometries. The calculated value of the average geometry was below the 25% percentile of all distributions except for the volume-averaged velocity and the airflow partitioning. While the cross-sectional areas calculated for the patient-specific geometries and the average geometry agreed well, especially in the anterior part of the nose, the minimal cross-sectional area of the average geometry was slightly larger than the median of all patient-specific geometries. As the minimal cross-sectional area is strongly correlated with the nasal resistance^31^, this might explain the deviations observed for the lateral and total resistances. Furthermore, most of the other investigated parameters are correlated with the patency of the nose, as a smaller minimal cross-sectional area will lead to an increased velocity within the vicinity of the isthmus nasi, where the predominant acceleration of airflow takes place. This increase of the airflow velocity will lead to increased wall shear stresses, as especially the inspiratory jet is directed towards the nasal septum^32,33^.

The isthmus nasi, which is the region of the nasal cavity featuring the minimal cross-sectional area, was defined as a key feature within the statistical shape model. This definition was based on visual examination by an ENT specialist. The minimal cross-sectional area was calculated perpendicular to streamlines derived from the numerical velocity fields^31^. Therefore, the definition of the isthmus nasi was done without any information on the relation between nasal geometry and airflow, whereas the minimal cross-sectional area is dependent on the resulting airflow. This might explain the deviation observed for the minimal cross-sectional area, while the remaining cross-sectional areas agreed well.

Another reason for the difference observed regarding the minimal cross-sectional area might be found in the method used to generate the statistical shape model. While the approach was successfully used for other complex geometries featuring high curvatures, as for example the pelvic bone, novel non-linear methods might result in a more accurate representation of the mean shape^34,35^.

As the relationship between the nasal cavity’s geometry and the airflow observed within is governed by a set of non-linear equations, the average geometry will not necessarily result in an airflow that equals the average of the individual airflows. However, if the average geometry already deviates in geometric aspects, this result is even more unlikely. The presented average geometry does not equal the average airflow observed within the 25 individual geometries.

However, the second question remains: Can the average geometry be used as generic geometry for investigations regarding healthy nasal airflow? While the integral measures calculated for the average geometry varied from the respective median values calculated for the patient-specific geometries, they lay within the distribution observed for those individual geometries. Therefore, there were also healthy geometries featuring lower resistances or smaller wall shear stress and velocity values than the average geometry. Furthermore, the values of lateral and total resistances as well as the minimal cross-sectional area calculated for the individual geometries as well as for the average geometry agree well with normative ranges of healthy nasal airflow that were identified by Borojeni *et al*. based on a sample of 47 symptom-free subjects^13^.

Additionally, comparison of the wall shear stress distribution of the averaged geometry and the median distribution calculated from the 25 patient-specific distributions revealed a good agreement. Regions with relatively high wall shear stresses were strikingly similar in both distributions. Only small deviations related to the shear layer, which is formed between the inspiratory jet and the large stationary vortex in the anterior upper part of the nasal cavity, were observed.

Also, comparison of static pressure distributions revealed good agreement. Here, the commonly observed steep pressure gradient at the isthmus nasi was observed. At this narrowest cross-section the airflow is accelerated and usually directed towards the middle meatus. However, the average total resistance of all 25 geometries was twice as large as the pressure drop calculated using the average geometry. This was found for inspiration and expiration independently. Therefore, only the patterns within the static pressure distributions were similar but not the absolute values observed. Interestingly, another similarity regarding the nasal resistance is, that it was larger during expiration than during inspiration for both, the average geometry as well as the average of all 25 subjects.

### Limitations

The findings of this study must be considered with respect to the limitations of the study’s design. First, the inclusion of subjects into the healthy cohort was based solely on the self-perceived perception of nasal airflow. As perception of nasal airflow is highly subjective, it is possible that some subjects within this study’s cohort show, at least, slight deviations toward impaired nasal breathing. While this would also affect the average geometry generated using the statistical shape model, it is difficult to estimate the bias introduced by this due to the non-linear relation between the nasal geometry’s anatomy and airflow. Additionally, this self-declaration of being symptom-free by the individual subjects was supported and made plausible through the clinical examination as well as the CT images. Both did not show any clear pathological findings.

Due to the averaging process, the average geometry features a fully symmetric and more regular geometry than the individual geometries. There are neither narrow slits nor extremely tortuous channels. In contrast, even in a healthy nasal cavity, asymmetries are usually observed due to minor septal deviations or the nasal cycle. Whereas the nasal cycle or minor septal deviations are shape-wise eliminated in the mean geometry, they might have an impact on the calculated airstream within the individual geometries, but without affecting the individuals’ nasal functions or perception. Already smaller deviations might decrease channel widths within the nasal cavity, resulting in increased local velocities and hence also increased wall shear stresses.

Gambaruto *et al*. also found that their average geometry, which was derived from three subjects featured less nasal resistance and less wall shear stresses than observed within the individual geometries^17^. They concluded, that the averaging of healthy nasal cavities did not result in a geometry representing the average airflow but an idealized airflow due to the regularization of the geometry.

In this study airflow during restful inspiration and expiration was calculated using a quasi-steady assumption. Recent studies indicate that this assumption must not be made when investigating particle deposition within the nasal cavity^36,37^. Thus, even though good agreements between the averaged distributions calculated in the average geometry were found, these findings might not be transferable to investigations regarding particle deposition.

The statistical shape model presented here was truncated at the nostrils as well as the pharynx and nasal sinuses as well as ethmoidal air cells were omitted. However, Taylor *et al*. showed that neglecting the ambient and the geometry of the face might result in changes for calculated wall shear stresses^32^. Due to the retrospective nature of this study, the face could not be reconstructed from all CT data sets. Furthermore, this truncation is common in other numerical investigations.

## Conclusion

We were able to generate an average geometry of the healthy nasal cavity and compare integral measures as for example the nasal resistance and wall shear stresses as well as the static pressure distributions calculated within this geometry against those parameters calculated for the 25 symptom-free subjects that were used for generation of the average geometry. The airflow observed within the average geometry did not equal the average airflow calculated using the individual simulations. Deviations found in the integral measures were more prominent than those in the spatially resolved distributions.

However, the mean geometry shows all common features of a healthy nasal cavity. Due to the averaging process, however, the overall shape is much more regularized than the patient-specific geometries. As the integral measures calculated for the average geometry were within the range defined by the patient-specific geometries and spatial distributions of wall shear stresses were similar, we consider the average geometry as a synthetic supplementary variant of an asymptomatic nasal cavity.

The projection of wall-bound values onto the average geometry of the nasal cavity seems promising. This would allow for a point-wise comparison of different symptomatic or non-symptomatic subjects. The latter we demonstrated in the study. In our future work, the SSM will be enhanced by implementing geometries of patients suffering from impaired nasal breathing.

## Data Availability

The original CT image data and segmentations are not publicly available due to the regulatory limitations. The average geometry of the statistical shape model as well as all static pressure and wall shear stress distributions and the integral parameters calculated for all individual cases are available via figshare: (10.6084/m9.figshare.9585410).
